# Toxicities and Cross-Resistance of Imidacloprid, Acetamiprid, Emamectin Benzoate, Spirotetramat, and Indoxacarb in Field Populations of *Culex quinquefasciatus* (Diptera: Culicidae)

**DOI:** 10.3390/insects13090830

**Published:** 2022-09-13

**Authors:** Muhammad Kamran, Sarfraz Ali Shad, Muhammad Binyameen, Naeem Abbas, Muhammad Anees, Rizwan Mustafa Shah, Abdulwahab M. Hafez

**Affiliations:** 1 Department of Entomology, Faculty of Agricultural Sciences and Technology, Bahauddin Zakariya University, Multan 60800, Pakistan; 2 Department of Plant Protection, College of Food and Agriculture Sciences, King Saud University, P.O. Box 2460, Riyadh 11451, Saudi Arabia; 3 Pest Warning and Quality Control of Pesticides Punjab, Lahore 5400, Pakistan

**Keywords:** southern house mosquito, vector-borne disease, resistance management, insecticide, lethal concentration

## Abstract

**Simple Summary:**

*Culex quinquefasciatus* is a major vector of several human and animal diseases. This species’ ability to develop resistance to synthetic insecticides can lead to failure to control it. Therefore, we conducted toxicity bioassays of imidacloprid, acetamiprid, emamectin benzoate, spirotetramat, and indoxacarb on five field populations of *Cx. Quinquefasciatus* from Pakistan. These five populations showed susceptibility to high resistance against imidacloprid, susceptibility to moderate resistance against acetamiprid, susceptibility to emamectin benzoate, susceptibility to spirotetramat, and low–high resistance against indoxacarb. Correlation analyses revealed a significant positive correlation between imidacloprid, acetamiprid, and spirotetramat median lethal concentration values, indicating the possibility of cross-resistance. Meanwhile, there were no significant correlations among the median lethal concentration values of other tested insecticides, indicating the possible absence of cross-resistance. Our findings provide a useful background for the public health authorities, medical entomologists, and pest managers to control *Cx. quinquefasciatus.*

**Abstract:**

*Culex quinquefasciatus* is a major vector of several pathogens and is capable of breeding in various aquatic habitats. The extensive and injudicious use of synthetic chemicals against the mosquito species has led to the problem of insecticide resistance. To explore this resistance in detail, toxicity bioassays of imidacloprid, acetamiprid, emamectin benzoate, spirotetramat, and indoxacarb were performed on five *Cx. quinquefasciatus* field populations from Pakistan in addition to a laboratory susceptible strain. Compared with the susceptible strain, results for the five *Cx. quinquefasciatus* field populations were as follows: susceptibility to high resistance against imidacloprid (resistance ratio (RR): 0.09–11.18), susceptibility to moderate resistance against acetamiprid (RR: 0.39–8.00), susceptibility to emamectin benzoate (RR: 0.002–0.020), susceptibility to spirotetramat (RR: 0.01–0.07), and low to high resistance against indoxacarb (RR: 3.00–118.00). Correlation analyses revealed a significant positive correlation between imidacloprid, acetamiprid, and spirotetramat median lethal concentration (LC_50_) values, indicating the possibility of cross-resistance. In contrast, there were no significant correlations between the LC_50_ values of other tested insecticides, indicating the possible absence of cross-resistance. These results can assist public health authorities, medical entomologists, and pest managers to manage the insecticide resistance of *Cx. quinquefasciatus* as well as the associated pollution and human health issues.

## 1. Introduction

The southern house mosquito *Culex quinquefasciatus* Say (Diptera: Culicidae) is a common species found in hot–humid and warm-temperate regions [1]. Sewerage lines, standing water, and ponds are suitable breeding habitats for *Cx. quinquefasciatus.* Humans and animals are affected severely by the biting of this species, often leading to disturbed sleep during rainy seasons [2]. More importantly, *Cx. quinquefasciatus* is a major vector of several pathogens that cause diseases, including yellow fever, filariasis, and Japanese encephalitis [3,4,5]. According to Michael and Bundy [6], about 91% of lymphatic filarial cases due to the nematode *Wuchereria bancrofti* Cobbold are vectored by *Cx. quinquefasciatus.*

To combat the effects of *Cx. quinquefasciatus*, synthetic insecticides from different chemical groups have been applied as part of various strategies, including their direct application to mosquito breeding sites, indoor residual spraying, and their use in insecticide-treated nets. Synthetic insecticides provide an adequate level of mosquito control due to their rapid knockdown effects. However, the development of resistance in mosquitoes against synthetic insecticides has brought into question their long-term sustainable efficacy [7,8,9]. Indeed, the application of insecticides at short and frequent intervals usually leads to the development of resistance to the applied active ingredients.

The control programs used against other mosquito species (as vectors of other important diseases) can play roles in the development of resistance in *Cx. quinquefasciatus*. For example, the spread of dengue fever in south and west Asia, including Pakistan and Saudi Arabia, since 2000 has forced governments to spray various insecticides into urban and semi-urban areas continuously to control dengue-spreading mosquitoes [10,11,12,13,14]. Thus, the probability that *Cx. quinquefasciatus* is exposed to the applied chemicals has increased due to the cohabitation of *Cx. quinquefasciatus* with dengue vector mosquitoes [15]. Overall, this insecticide use practice poses a serious threat to the success of insecticide-based vector management as well as causes environmental pollution and human health risks [16,17,18].

Agrochemicals can also contribute to the selection of resistance genes in mosquitoes [19,20,21]. Many mosquito species are known to use insecticide-contaminated swamps and wetlands in agroecosystems as their breeding habitats [22]. Increased exposure to insecticides may increase the risk of resistance development in mosquitoes if such exposure is not managed properly. Therefore, continuous resistance monitoring is needed to aid the development of novel and environmentally friendly control strategies. Indeed, such monitoring provides not only records of the spatial and temporal variation in populations’ responses to insecticides but also early warnings of insect resistance. It is important to select insecticides that show efficacy in controlling mosquitoes while also reducing pollution [23]. Insecticide resistance has been recorded in *Cx. quinquefasciatus* in several previous studies [9,24,25]. To date, *Cx. quinquefasciatus* has shown resistance to >40 active ingredients belonging to different chemical groups, including new chemical insecticides, worldwide [26]. In Pakistan, toxicities and laboratory-selected resistance to insecticides have been previously reported in *Cx. quinquefasciatus* [27,28,29]. However, levels of field-evolved resistance in *Cx. quinquefasciatus* to insecticides have not been reported from Punjab, Pakistan.

Therefore, the present study was conducted to investigate the susceptibility/resistance status of *Cx. quinquefasciatus* field populations (collected from five different localities in Punjab, Pakistan) in relation to five common chemical insecticides: imidacloprid, acetamiprid, emamectin benzoate, spirotetramat, and indoxacarb. Overall, the aim was to provide data that would aid the design of future resistance management strategies and improve *Cx. quinquefasciatus* management. The results of this study revealed possible cross-resistance among some of the tested insecticides but not among others; thus, the findings may be useful for the continuous management of insecticide resistance in *Cx. quinquefasciatus*.

## 2. Materials and Methods

### 2.1. Insect Collection and Rearing

*Culex quinquefasciatus* larvae and pupae were collected randomly from stagnant contaminated water using a plastic dipper. Collections were undertaken in semi-urban settings near agricultural sites in five different districts in Punjab, Pakistan: Multan (30.1978° N, 71.4697° E), Khanewal (30.3030° N, 71.9309° E), Muzaffargarh (30.0703° N, 71.1933° E), Bahawalpur (29.3956° N, 71.6836° E), and Lodhran (29.5333312° N, 71.6333308° E). A laboratory susceptible strain (named SUS-ST) was used as a reference for comparison with the field populations. The SUS-ST strain has been maintained since 1990 without exposure to any chemicals at the Pesticides and Environmental Toxicology Laboratory, Department of Plant Protection, College of Food and Agriculture Sciences, King Saud University, Riyadh, Saudi Arabia. Previously, the SUS-ST strain served as a reference strain in the work of Hafez and Abbas [9]. All collected populations were named with respect to their locations as follows: Multan, MLT-POP; Khanewal, KHN-POP; Muzaffargarh, MZG-POP; Bahawalpur, BWP-POP; and Lodhran, LOD-POP. All populations were reared under controlled conditions: 28 °C ± 2 °C, 65 ± 5% relative humidity, and a 12/12 h (light/dark) photoperiod. The collected larvae were separated from their water source using plastic suckers and placed in 1200 mL plastic containers filled with fresh tap water (1000 mL). Ground artificial fish food (CAPRIMA, Indonesia) was provided to the larvae in each container according to their population density. After the larval stage was complete, pupae were moved to plastic cups containing 120 mL of tap water, which were placed in aerated plastic cages until adult emergence. Subsequently, a cotton wick (3–4 cm in length) was dipped in a 10% sugar solution and provided to the adults as a diet source in a 10 mL plastic vial. The cotton wick was hydrated with sugar solution daily, and each wick was replaced every 3 days. At 5–6 days after adult emergence, a pigeon was placed in the adult cage at night for 5–6 h as a blood source for female mosquitoes. A 120 mL plastic cup filled with tap water was also placed in the cage housing adults as an egg-laying site. Laid eggs were collected from the water surface using a camel hair brush and placed in a plastic container for hatching. Neonates were fed with the fish food described above until the fourth instar, and these individuals were named the F_1_ generation and used in bioassays [30].

### 2.2. Female Mosquitoes’ Blood-Feeding

For blood-feeding mosquitoes, pigeons were kept in pairs in cages in the animal care facility, Bahauddin Zakariya University, Multan, and provided with water and cereal grains ad libitum. Every pigeon was fed cereals and water in the facility for fifteen days before being used in an experiment. When serving as a blood source for female mosquitoes, each pigeon was restrained with rubber straps and cotton around the legs and body then placed on the screen top of the mosquito-rearing cage for maximum 45 min. Each pigeon was exposed to approximately 50–100 mosquitoes during each feeding. To minimize any distress to these pigeons, individual pigeons were exposed to blood feeding only once a month and were released after three months of being used in any experiment.

### 2.3. Insecticides

The five commercially available chemical insecticides used in the bioassays against *Cx. quinquefasciatus* larvae are shown in Table 1.

### 2.4. Bioassays

The toxicity of insecticides against the fourth instar larvae of *Cx. quinquefasciatus* was evaluated using a bioassay protocol described by the World Health Organization [31] under the aforementioned conditions. Using the serial dilution method, five concentrations of each insecticide were produced for each bioassay, and each concentration was replicated three times. Thus, 30 fourth instar larvae were used to test 1 concentration (10 larvae/replicate), and 150 larvae were used in each bioassay. The control group consisted of 30 larvae and was tested with tap water only. Larvae were provided with artificial fish food ad libitum. Mortality was assessed after 48 h of exposure, and the larvae were scored as dead if they were unable to move even after the container was tapped.

### 2.5. Statistical Analysis

Mortality data were assessed using probit analysis [32] via Probit Analysis software [33], and LC_50_ values were calculated with 95% confidence intervals (CIs). LC_50_ values were considered significantly different when their 95% CIs did not overlap [34]. Resistance ratios (RR_50_) were calculated by dividing the LC_50_ value of each insecticide in each field population by its LC_50_ value in the susceptible strain. Insecticide resistance levels were classified using the following criteria: susceptibility or low resistance (resistance ratio (RR) < 5), moderate resistance (RR = 5–10), and high resistance (RR > 10) [35]. Correlations between LC_50_ values were calculated using Pearson correlation via Statistix 8.1 [36].

## 3. Results

### 3.1. Resistance of Cx. quinquefasciatus to the Neonicotinoids Imidacloprid and Acetamiprid

The LC_50_ value of imidacloprid in SUS-ST was 0.011 µg/mL (95% CI: 0.007–0.015). The LC_50_ values of imidacloprid were 0.001–0.123 µg/mL in the *Cx. quinquefasciatus* field populations, with KHN-POP showing high resistance (RR = 11.18) and MLT-POP, MZG-POP, LOD-POP, and BWP-POP showing low resistance/susceptibility (RR = 0.09, 0.64, 2.82, and 1.27, respectively) to imidacloprid (Table 2).

The LC_50_ value of acetamiprid for SUS-ST was 0.013 µg/mL (95% CI: 0.010–0.017). The LC_50_ values of acetamiprid were 0.005–0.104 µg/mL in the *Cx. quinquefasciatus* field populations. Most field populations showed susceptibility/low resistance (RR = 1.87, 0.39, 3.00, and 1.62 for MLT-POP, MZG-POP, LOD-POP, and BWP-POP, respectively) to acetamiprid, with the exception of KHN-POP, which showed moderate resistance (RR = 8.00) (Table 2).

### 3.2. Resistance of Cx. quinquefasciatus to the Avermectin Emamectin Benzoate

The LC_50_ value of emamectin benzoate in SUS-ST was 0.082 µg/mL (95% CI: 0.031–0.132). The LC_50_ values of emamectin benzoate were 0.0002–0.0020 µg/mL in the *Cx. quinquefasciatus* field populations with RRs of 0.002–0.020. All field populations were more susceptible to emamectin benzoate compared with SUS-ST (nonoverlapping 95% CIs, Table 3).

### 3.3. Resistance of Cx. quinquefasciatus to the Tetramic Acid Derivative Spirotetramat

The LC_50_ value of spirotetramat in SUS-ST was 13.46 µg/mL (95% CI: 8.37–34.94). The LC_50_ values of spirotetramat in the *Cx. quinquefasciatus* field populations were 0.068–0.991 µg/mL, and the RRs were 0.01–0.07. All *Cx. quinquefasciatus* field populations were more susceptible to spirotetramat compared with SUS-ST (nonoverlapping 95% CIs, Table 3).

### 3.4. Resistance of Cx. quinquefasciatus to the Oxadiazine Indoxacarb

The LC_50_ value of indoxacarb in SUS-ST was 0.002 µg/mL (95% CI: 0.001–0.004). The LC_50_ values of indoxacarb in the *Cx. quinquefasciatus* field populations were 0.006–0.236 µg/mL, with KHN-POP and BWP-POP showing high resistance (RR = 13.00 and 118.00, respectively), MLT-POP and LOD-POP showing moderate resistance (RR = 9.50 and 8.50, respectively), and MZG-POP showing susceptibility/low resistance (RR = 3.00) to indoxacarb (Table 3).

### 3.5. Pair-Wise Comparisons of the Tested Chemical Insecticides

The toxicity of imidacloprid was significantly positively correlated with the toxicities of acetamiprid and spirotetramat and nonsignificantly positively correlated with the toxicity of emamectin benzoate; however, imidacloprid toxicity was negatively correlated with indoxacarb toxicity. The toxicity of acetamiprid was significantly positively correlated with that of spirotetramat, nonsignificantly positively correlated with that of emamectin benzoate, and nonsignificantly negatively correlated with that of indoxacarb. The toxicity of emamectin benzoate was nonsignificantly positively correlated with the toxicities of spirotetramat and indoxacarb, whereas the toxicity of indoxacarb was nonsignificantly positively correlated with that of spirotetramat (Table 4).

## 4. Discussion

When disease-transmitting mosquitoes must be controlled quickly, such as during outbreaks of *Cx. quinquefasciatus*-associated illnesses, chemicals are employed to reduce the mosquito population size rapidly. However, the development of resistance to insecticides due to such practices affects the efficacy of the insecticides in vector control and may even facilitate the further spread of disease [37]. Thus, it is necessary to evaluate the susceptibility/resistance status of insect vectors to commonly used insecticides and select the most appropriate and effective insecticide for use in practice [9]. Therefore, the current imidacloprid, spirotetramat, emamectin benzoate, acetamiprid, and indoxacarb susceptibility/resistance status of five *Cx. quinquefasciatus* populations was evaluated in the present study with the aim of improving *Cx. quinquefasciatus* control strategies.

All tested *Cx. quinquefasciatus* strains showed different RRs for imidacloprid ranging from 0.09- to 11.18-fold. Different resistance levels against imidacloprid have been reported previously in various medically important pests found worldwide, including *Cx. quinquefasciatus* [38], *Aedes aegypti* L. [39], and *Musca domestica* L. [40,41]. In the present study, changes in acetamiprid susceptibility were observed in KHN-POP (moderate resistance; RR = 8.00-fold) and LOD-POP (low resistance; RR = 3.00-fold), whereas all other tested populations remained susceptible to this insecticide. Acetamiprid resistance may have arisen in KHN-POP due to its extensive use in the Khanewal district to prevent pest attacks on the main crops (i.e., cotton and rice), which may have led to the selection of resistance genes in *Cx. quinquefasciatus*, as has been reported in other pest species [42]. For example, resistance to acetamiprid has been reported in *M. domestica* [41], *Blattella germanica* L. [43], and *Dysdercus koenigii* Fabricius [44]. Moreover, laboratory-induced resistance to acetamiprid was reported in *Ae. aegypti* [45].

High susceptibility to spirotetramat was observed in all *Cx. quinquefasciatus* populations in the present study, suggesting that spirotetramat can provide effective control during *Cx. quinquefasciatus* outbreaks. In contrast, *M. domestica* has developed resistance to some keto-enols, such as spiromesifen (from the same chemical group as spirotetramat) [46]. In the current study, all field *Cx. quinquefasciatus* populations showed high susceptibility to emamectin benzoate. Resistance to emamectin benzoate has been documented in *D. koenigii* [44], *M. domestica* [47], and *Aedes albopictus* Skuse [48]; however, the present results showed that emamectin benzoate could be used to control *Cx. quinquefasciatus*. In tests of indoxacarb, two of the five *Cx. quinquefasciatus* populations exhibited a high level of resistance, whereas two populations showed moderate resistance and one population showed low resistance. Previously, Khan et al. [47] and Abbas et al. [49] reported different levels of indoxacarb resistance in *M. domestica* populations collected from different locations in southern Punjab, Pakistan. Resistance to indoxacarb in *M. domestica* was also reported by Shono et al. [50]. In addition, high levels of indoxacarb resistance were also found in *Ae. albopictus* collected from different localities in Pakistan [48].

The insecticides to which *Cx. quinquefasciatus* were susceptible or showed low resistance could be effective chemical tools in the control and management of this medically important vector. However, high levels of resistance to indoxacarb were found in two *Cx. quinquefasciatus* populations, possibly due to the exposure of these populations to different selection pressures, cross-resistance mechanisms among chemicals, injudicious use of the pesticides in vector management programs, and/or the involvement of resistance mechanisms (e.g., elevated enzymatic detoxification or target site insensitivity) [51].

Moreover, it is possible that *Cx. quinquefasciatus* larvae have evolved resistance through their exposure to residues of insecticides applied in agriculture that drift into mosquito breeding sites [20]. Such insecticide residues have lethal effects on the larvae of some mosquito populations but exert selection pressure on other populations, leading to the emergence of resistant populations [52]. Mosquitoes may also be exposed to agrochemicals during their foraging flights between breeding habitats, e.g., when they rest on treated crops [48]. Such exposures of vector insects to agrochemicals influence resistance development [53]. Overall, the present results suggest that *Cx. quinquefasciatus* was generally susceptible to the tested active ingredients in relatively new classes of chemical insecticides, although a few populations exhibited elevated resistance. Our data also highlight the need to consider the history of the chemicals being used in agricultural and nonagricultural areas prior to implementing vector management measures to avoid resistance development and control the pest species successfully.

The use of many agrochemicals overlaps with the use of chemicals in vector management programs by public health agencies, which further complicates insecticide resistance management. According to the present results, *Cx. quinquefasciatus* might have developed resistance to insecticides due to possible cross-resistance mechanisms among the various agrochemicals. Pair-wise correlation coefficient analysis of the LC_50_ values of the tested insecticides in *Cx. quinquefasciatus* field populations revealed that most of the insecticides were positively correlated in terms of their toxicities, indicating the possibility of an underlying cross-resistance mechanism. For example, we found highly significant and positive correlations between acetamiprid and imidacloprid or spirotetramat and between imidacloprid and spirotetramat, suggesting the existence of cross-resistance and the presence of a common mechanism or multiple resistance mechanisms for insecticides with different modes of action. Consistent with our results, highly significant positive correlations were reported between insecticides with the same mode of action [42] and different modes of action [54]. We also detected a lack of cross-resistance among various insecticides in the present study, suggesting that these chemicals could be used alternatively to manage resistance development over a long period. However, any assumption based on the findings of the current study should be limited by the spatial frame of collection sites of these populations, and any kind of generalization regarding the cross-resistance between the tested chemicals should be avoided. Therefore, further studies are needed to confirm the existence of cross-resistance between these chemicals and to fully understand their biological causes.

In summary, assessments of resistance status can help select compounds with promising results to target *Cx. quinquefasciatus*, which can help minimize the spread of diseases due to this vector. The larvae of *Cx. quinquefasciatus* from different localities showed different levels of resistance against all tested insecticides, highlighting the poor systematic management practices used in Pakistan. Early detection of elevated resistance levels can prompt the public health authorities, medical entomologists, scientists, and pest managers involved in vector control activities to implement appropriate measures to control *Cx. quinquefasciatus*. Importantly, the insecticides to which *Cx. quinquefasciatus* showed susceptibility/low resistance have the potential for high efficacy in the control of this disease vector. Correlation analyses revealed a highly significant and positive correlation between acetamiprid and imidacloprid or spirotetramat, suggesting that cross-resistance to these insecticides may have arisen. Awareness of cross-resistance to the same or different synthetic compounds used as larvicides against mosquito populations can enable professionals to control this pest more effectively. Overall, our results can help interrupt resistance development in *Cx. quinquefasciatus* and limit the spread of mosquito-borne diseases.

## Figures and Tables

**Table 1:** List of the insecticides used in the *Culex quinquefasciatus* bioassays.

Chemical Class	Active Ingredient	Trade Name	Formulation ^1^	Manufacturer	IRAC ^2^ Mode of Action
Neonicotinoid	Imidacloprid	Confidor	20% SL	Bayer Crop Sciences	Nicotinic acetylcholine receptor competitive modulators (Group no.: 4A)
Neonicotinoid	Acetamiprid	Mospilan	20% SP	Arista Life Sciences	
Avermectins	Emamectin benzoate	Proclaim	1.9% EC	Syngenta	Glutamate-gated chloride channel allosteric modulators (Group no.: 6)
Tetramic acid derivatives	Spirotetramat	Movento	24% SC	Bayer Crop Sciences	Inhibitors of acetyl CoA carboxylase (Group no.: 23)
Oxadiazines	Indoxacarb	Steward	15% SC	DuPont	Voltage-dependent sodium channel blockers (Group no.: 22A)

^1^ SL: Soluble liquid, SP: Soluble powder, EC: Emulsifiable concentrate, and SC: Suspension concentrate. ^2^ Insecticide resistance action committee.

**Table 2:** Resistance levels of *Culex quinquefasciatus* to imidacloprid and acetamiprid.

Insecticide	Population	N ^a^	LC_50_^b^ (μg/mL)	95% CI ^c^ (μg/mL)	Slope ± SE	χ^2 d^	df ^e^	*P*	RR ^f^
Imidacloprid	SUS-ST	180	0.011	0.007–0.015	1.61 ± 0.28	0.25	3	0.99	1.00
	MLT-POP	180	0.001	0.000–0.001	0.96 ± 0.16	1.88	3	0.76	0.09
	MZG-POP	180	0.007	0.004–0.010	1.67 ± 0.32	2.2	3	0.70	0.64
	LOD-POP	180	0.031	0.024–0.040	2.26 ± 0.32	3.13	3	0.54	2.82
	KHN-POP	180	0.123	0.098–0.153	2.68 ± 0.36	4.71	3	0.32	11.18
	BWP-POP	180	0.014	0.010–0.018	2.23 ± 0.36	1.48	3	0.83	1.27
Acetamiprid	SUS-ST	180	0.013	0.010–0.017	2.24 ± 0.32	4.10	3	0.39	1.00
	MLT-POP	180	0.023	0.017–0.029	3.85 ± 0.76	1.99	3	0.74	1.87
	MZG-POP	180	0.005	0.004–0.007	1.95 ± 0.31	2.58	3	0.63	0.39
	LOD-POP	180	0.039	0.032–0.049	2.87 ± 0.38	2.23	3	0.69	3.00
	KHN-POP	180	0.104	0.084–0.131	2.66 ± 0.36	7.25	3	0.12	8.00
	BWP-POP	180	0.021	0.015–0.027	2.28 ± 0.35	1.17	3	0.88	1.62

^a^ Number of tested larvae. ^b^ Median lethal concentrations. ^c^ Confidence intervals of the LC_50_ values. ^d^ Chi-square. ^e^ Degrees of freedom. ^f^ Resistance ratio (was calculated by dividing the LC_50_ value of each insecticide in each field population by its LC_50_ value in the susceptible strain).

**Table 3:** Resistance levels of *Culex quinquefasciatus* to emamectin benzoate, spirotetramat, and indoxacarb.

Insecticide	Population	N ^a^	LC_50_^b^ (μg/mL)	95% CI ^c^ (μg/mL)	Slope ± SE	χ^2 d^	df ^e^	*P*	RR ^f^
Emamectin benzoate	SUS-ST	180	0.082	0.031–0.132	1.18 ± 0.28	0.92	3	0.92	1.00
	MLT-POP	180	0.0002	0.0001–0.0009	0.92 ± 0.26	0.65	3	0.96	0.002
	MZG-POP	180	0.001	0.000–0.001	1.55 ± 0.28	1.49	3	0.83	0.01
	LOD-POP	180	0.002	0.002–0.003	2.00 ± 0.30	4.85	3	0.30	0.02
	KHN-POP	180	0.002	0.001–0.002	2.30 ± 0.37	0.82	3	0.94	0.02
	BWP-POP	180	0.002	0.001–0.002	2.06 ± 0.33	1.52	3	0.82	0.02
Spirotetramat	SUS-ST	180	13.46	8.37–34.94	1.12 ± 0.27	0.84	3	0.93	1.00
	MLT-POP	180	0.068	0.036–0.102	1.16 ± 0.21	6.06	3	0.19	0.01
	MZG-POP	180	0.124	0.083–0.166	1.81 ± 0.31	3.06	3	0.55	0.01
	LOD-POP	180	0.070	0.053–0.089	2.19 ± 0.32	3.29	3	0.51	0.01
	KHN-POP	180	0.991	0.774–1.271	2.26 ± 0.32	6.56	3	0.16	0.07
	BWP-POP	180	0.406	0.305–0.532	1.98 ± 0.30	0.71	3	0.95	0.03
Indoxacarb	SUS-ST	180	0.002	0.001–0.004	1.48 ± 0.26	0.23	3	0.99	1.00
	MLT-POP	180	0.019	0.013–0.027	1.44 ± 0.22	4.77	3	0.31	9.50
	MZG-POP	180	0.006	0.005–0.008	2.11 ± 0.31	1.23	3	0.87	3.00
	LOD-POP	180	0.017	0.012–0.022	2.09 ± 0.32	3.01	3	0.56	8.50
	KHN-POP	180	0.026	0.018–0.034	2.10 ± 0.35	1.85	3	0.76	13.00
	BWP-POP	180	0.236	0.181–0.300	2.26 ± 0.32	3.13	3	0.54	118.00

^a^ Number of tested larvae. ^b^ Median lethal concentrations. ^c^ Confidence intervals of the LC_50_ values. ^d^ Chi-square. ^e^ Degrees of freedom. ^f^ Resistance ratio (was calculated by dividing the LC_50_ value of each insecticide in each field population by its LC_50_ value in the susceptible strain).

**Table 4:** Pair-wise correlation coefficients of the LC_50_ values of insecticides against *Culex quinquefasciatus*.

Insecticide	Imidacloprid	Acetamiprid	Emamectin Benzoate	Spirotetramat
Acetamiprid	0.98 *			
Emamectin benzoate	0.55 ^ns^	0.48 ^ns^		
Spirotetramat	0.91 *	0.87 *	0.53 ^ns^	
Indoxacarb	−0.18 ^ns^	−0.19 ^ns^	0.41 ^ns^	0.15 ^ns^

* Significant (*p* ≤ 0.05); ^ns^ nonsignificant.

## Data Availability

The data presented in this study are available from the corresponding author on a reasonable request.
